# Personalized Coaching via Texting for Behavior Change to Understand a Healthy Lifestyle Intervention in a Naturalistic Setting: Mixed Methods Study

**DOI:** 10.2196/47312

**Published:** 2023-11-15

**Authors:** Charlotte Bäccman, Linda Bergkvist, Erik Wästlund

**Affiliations:** 1 Service Research Center (CTF) Karlstad University Karlstad Sweden

**Keywords:** digital health intervention, behavior change, personalized SMS coaching, Capability, Opportunity, Motivation–Behavior, COM-B, physical activity, mixed methods design, mobile phone

## Abstract

**Background:**

Digital health interventions, such as personalized SMS coaching, are considered affordable and scalable methods to support healthy lifestyle changes. SMS, or texting, is a readily available service to most people in Sweden, and personalized SMS coaching has shown great promise in supporting behavior changes.

**Objective:**

This study aims to explore the effectiveness of highly personalized SMS coaching for behavior change according to the Capability, Opportunity, Motivation–Behavior (COM-B) model on a sample of physically inactive adults in a nonprofit fitness organization in Sweden.

**Methods:**

The study used a mixed methods design in which clients acted as their own controls. The participants were clients (n=28) and fitness consultants (n=12). Three types of data were collected: (1) quantitative data at baseline and after the SMS intervention and the waitlist from the clients, (2) qualitative data from semistructured interviews with the fitness consultants, and (3) pseudonymized texting conversations between the fitness consultants and clients.

**Results:**

Overall, the results showed that personalized SMS coaching was effective in supporting the clients’ behavior changes. The quantitative analysis showed how the clients’ capabilities (Cohen *d*=0.50), opportunities (Cohen *d*=0.43), and relationship with the fitness consultants (Cohen *d*=0.51) improved during the SMS intervention in comparison with baseline. Furthermore, the qualitative analysis revealed how personalized texts added value to existing work methods (eg, increasing continuity and flexibility) and how the relationship between the clients and fitness consultants changed during the intervention, which helped motivate the clients.

**Conclusions:**

Personalized SMS coaching is an effective method for supporting healthy behavior changes. The human connection that emerged in this study needs to be further explored to fully understand the effectiveness of a digital health intervention.

## Introduction

### Background

A physically active lifestyle contributes to a healthy and long life [1-3]. Being physically active is partly a choice and partly a consequence of external circumstances such as area of residence [4]. For example, “girls, women, older adults, underprivileged groups, and people with disabilities and chronic diseases” are among the most sedentary owing to limited access to affordable and safe opportunities for physical activities [5]. Thus, to increase equal opportunity and motivation for physical activity, both individual and societal aspects need to be addressed [4-6]. However, this study is limited to the individual perspective and the acknowledgment that, regardless of their personal circumstances, many people want to become healthier.

Digital health innovation can reduce health inequities [7]. Mönninghoff et al [8] claimed that digital innovations can be particularly useful for persons at risk and populations that are sick and present an affordable alternative for larger populations, whereas Western et al [9] claimed that those with low socioeconomic status are still left behind. Regardless, in 2021, the World Health Organization (WHO) issued the “Global strategy on digital health 2020-2025” [10] partly on the assumption that “digital innovations [can] promote and support people of all ages to be more active” [5]. Undoubtedly, finding effective and intersectional digital health innovations to support healthy behavior changes is of great importance.

### Changing Behaviors

Theories on learning and behavior change acknowledge the importance of both internal and external factors for behaviors. The classical social cognitive theory by Bandura [11] makes 3 basic postulations: first, people can learn through observation (ie, imitation); second, learning is influenced by mental states (eg, self-efficacy); and third, learning does not suffice for behavior change. Whether the observed behavior is adopted or not will depend on the observed reactions from the surroundings and feedback from imitating the behavior [11,12]; thus, the social cognitive theory shows the constant interaction between the person and the environment and the several critical points for disruption from observation to imitation.

Similarly, several theories that aim to describe the process of behavior change have acknowledged the interaction between the person and the environment [4,6,13,14]. One model that has combined several theories and techniques of behavior change is the Capability, Opportunity, Motivation–Behavior (COM-B) model [15,16]. The name of the model is an acronym for the 3 components deemed necessary for behavior change to occur, that is, capability, opportunity, and motivation [15]*.* Capability is the individual’s psychological and physical capacity to perform the desired behavior (eg, how to eat healthy or being aware that physical exercise is good for health); opportunity refers to the external conditions, such as living in a safe environment to exercise in or being able to afford healthy food, that promote desired behavior; and motivation includes all processes that direct behavior (eg, feelings and attitudes toward the behavior or the self). All 3 must occur simultaneously for the desired behavior to occur [15]. In other words, even if a person wants to change (ie, motivation) and knows what to do (ie, capability), their living situation can upturn the execution of the new behavior (ie, opportunity). In addition, behaviors are never fully extinguished, that is, the old behavior is only dormant and needs to be suppressed across a variety of situations, whereas the new behavior needs to be activated [13]. Thus, behavior change is unstable and can easily be disturbed [13]. In sum, changing a behavior is a challenge that goes beyond a willingness to change; opportunities for change and strategies for sustaining new, healthy behaviors should be determined on a daily basis [17,18].

There are numerous ways to support healthy behavior changes. For example, a systematic review and meta-analysis by Howlett et al [19] showed that behavior change techniques (BCTs) such as giving feedback about the behavior or strengthening capability and motivation were successful in helping adults who are inactive increase their level of physical activity. Samdal et al [20] obtained a similar result in a review and meta-analysis of physical activity and healthy eating among overweight or obese people. They pointed out that BCTs that help the person’s capabilities in combination with a person-centered method that enhances motivation are the driving principle for behavior changes in the reviewed studies. Their categorization of BCTs in the review was based on a taxonomy by Michie et al [16]. Bringing together the accumulated evidence and knowledge from years of research on BCTs in the context of fast-paced digitalization is a promising path forward [21].

### Digital Health Interventions for Behavior Change

Numerous digital health interventions (DHIs) have been designed to support behavioral changes [22]. DHI are health interventions delivered via digital devices or technologies, for example, smartphones, digital platforms, or SMS [23]. Most DHIs are designed to facilitate behavior changes using existing BCTs [24,25], where different types of feedback are the most commonly used strategy [21,26,27]. However, research on the effectiveness of DHIs and their effect on behavior changes is still unclear [21,28].

So far, the more personalized the DHI, the more effective it is for behavior change. Personalized DHIs—that is, interventions adapted to individual needs—“improve prevention, self-management and access to healthcare” [29]. For example, Wannheden et al [30] explored how self-monitoring and communication with health care via digital technology satisfy or frustrate basic psychological needs and found that individual preferences vary and that personalization of digital technological tools is essential. However, of the numerous digital health applications [31], few seem to offer personalized support.

Willcox et al [32] argued for the use of SMS for personalization; it is a widely adopted and inexpensive mobile function. The effectiveness of different types of SMS support has been studied quite extensively in different settings—for example, smoking cessation, medication adherence, and self-management of long-term conditions and health, including diabetes and weight loss [32,33]—and with varying degrees of personalization from simple to more tailored SMS prompts [34-37]. In the meta-analysis with a randomized control trial by Head et al [38], the SMS was automated and tailored to the participant’s characteristics (eg, age, health status, or motivation) or targeted the intended behavior (eg, smoking cessation). Most studies combined automated SMS with at least 1 more intervention (websites, print materials, or human counselors). Only half of the included studies were theory-based interventions, and personalization across studies varied considerably. “Personalization” was defined based on whether the SMS included the participant’s name or not. The result showed that studies that tailored the SMS according to demographics and psychosocial factors (ie, motivation or self-efficacy) and requested frequency were more effective than studies using only targeted SMS and a set SMS frequency [38]. Head et al [38] speculated whether the tailored SMS indicates a “social presence” that augments the intervention [39].

The importance of the relationship between the therapist and the client is well known in psychology [40]. A systematic review conducted by Chatterjee et al [21] revealed that digital interventions held promise for effectiveness, with a particular emphasis on the significance of personalization and tailoring for success. Among these interventions, video-based approaches were found to be the most well received. Thus, the user perspective needs to be considered, and the authors pushed for “in-depth qualitative research to gradually improve interventions to satisfy users” [21]. They also stressed the importance of knowing the patient “to ensure both relationship communication and goal-oriented coaching” [21]. Taken together, there is substantial evidence supporting the effectiveness of using personalized SMS as a DHI to facilitate behavior change. However, additional research is required to delve into the individual and social dimensions of DHIs.

### Objectives

The aim of this study was 2-fold: to test the effectiveness of theory-driven personalized SMS coaching in a naturalistic setting in Sweden using the COM-B model [15] and to explore user experiences of personalized SMS coaching at a Swedish nonprofit fitness organization; here, the users are both fitness consultants and clients. This mixed methods study used 3 types of data: quantitative self-reported data from the clients, pseudonymized SMS conversations between the fitness consultants and clients from the intervention, and qualitative interviews with the fitness consultants. The purpose of the mixed methods design is to (1) investigate the effect of personalized SMS coaching and its underlying process and (2) to further explain and validate these findings with follow-up interviews.

Personalized SMS is defined as content and frequency that are highly tailored to a client’s specific needs and wants.

## Methods

### Study Setting

Fitness in Värmland (FiV; Friskvården i Värmland in Swedish) is a nonprofit organization in a rural region in middle western Sweden with a longstanding mission to support the citizens in the region in making changes regarding unhealthy dietary habits and sedentary lifestyles. Approximately 20 fitness consultants use individual health coaching, group training, or diet courses to support their clients’ lifestyle changes. The main focus is to help clients find and sustain motivation to become healthier; for this, motivational interviewing (MI) and SMART (specific, measurable, attainable, realistic, timely) goal setting are used. MI is a person-centered conversation methodology that aims to enhance individuals’ decisions to initiate behavior changes [41]. According to MI, ambivalence is a necessary drive for change and starts when a person starts to question their current way of living, and MI aims to help the client formulate their own drive for change [41]. Therefore, the fitness consultants address both the aspects of capability and motivation.

The vast majority of FiV clients (75%) are prescribed to increase their physical activity by licensed medical staff (eg, physicians, nurses, and physiotherapists) to prevent or reduce symptoms of a clinical diagnosis such as diabetes, heart and vascular diseases, and anxiety. The remaining 25% voluntarily seek help to achieve healthier lifestyle choices. Most clients are female (65%), aged >40 years (77%), and many persons have comorbidities (the percentages are from 2019 and were provided by FiV).

When the clients first come to 1 of the 16 FiV centers in the region, they are tested for basic health indicators (eg, weight, aerobic fitness, and physical and dietary habits) [42]. Some of these tests were included in this study and are described in more detail in the Materials section. The fitness consultants also start an MI-based dialogue (health talks) with the clients about lifestyle choices and possible changes. The health talks are then offered on a regular basis, often every fourth to sixth week.

### Design

This study used a within-subject design with an ABBA design, set in a naturalistic setting using completion data only, that is, the study only included participants who followed through the intervention, and no imputations were made for missing data. Using an ABBA design offers several advantages. It is straightforward to put into practice, ensures robust experimental control, counters order effects, and acknowledges that certain behaviors such as acquiring new skills may not be reversible [43]. The recruited clients were randomized to start with either the 8-week SMS intervention (condition A) or placed on the waiting list (condition B). Table 1 presents the overview and timeline. The clients who started with the SMS intervention (ie, AB group) were placed on the waitlist after the 8-week intervention, and the clients who started on the waitlist (ie, BA group) were given the intervention after the 8-week waitlist. Thus, the clients acted as their own controls (Table 1). As Table 1 shows, the quantitative self-reported data from the clients were collected on 3 different occasions, time 1 (T1) to time 3 (T3), where the order of the interventions varied.

**Table 1:** Study design and measurements from time 1 (T1) to time 3 (T3) for the participating clients (n=28).

Group	T1 (week 0; baseline)	Weeks 1-8	T2^a^ (week 8)	Weeks 9-16	T3 (week 16)
AB^b^	SMS guide Lifestyle questions Physical fitness FiV^c^ questionnaire	A^d^: SMS coaching	Physical fitness Lifestyle questions FiV questionnaire SMS evaluation	B^e^: waitlist	Physical fitness Lifestyle questions FiV questionnaire Interview by fitness consultant Interview with fitness consultants
BA^f^	SMS guide Lifestyle questions Physical fitness FiV questionnaire	B: waitlist	Physical fitness Lifestyle questions FiV questionnaire	A: SMS coaching	Physical fitness Lifestyle questions FiV questionnaire SMS evaluation Interview by fitness consultant Interview with fitness consultants

^a^T2: time 2.

^b^AB: the clients who started with the SMS intervention and after this was on waitlist.

^c^FiV: Fitness in Värmland.

^d^A: SMS intervention phase.

^e^B: waitlist phase.

^f^BA: the clients who started with the waitlist and after this was given the SMS intervention.

### Ethical Considerations

This study follows the ethical principles for research involving human subjects as stated by the World Medical Association, and all participants gave their informed consent to participation. The study was approved by an ethical advisor at Karlstad University (HS 2020/102). No clinical trial registration has been made. All participants provided their informed consent and were advised that all participation was voluntary. If they chose not to participate or later wanted to opt out, they were informed that this would not affect their regular support from the fitness consultants. All participation data from the clients was anonymous to the researchers. The researchers only had contact with the fitness consultants and all their data has been pseudonymized (deidentified). No compensation was offered to the participants, clients, or fitness consultants.

The mixed method used three types of data: (1) quantitative self-reported data, (2) SMS conversations between the fitness consultants and clients during the intervention, and (3) qualitative interviews with the fitness consultants after the intervention. Using different data, we accumulated different viewpoints on the effects of personalized SMS on behavior changes and the participants’ experiences from SMS coaching. The integration of quantitative self-report data and qualitative SMS conversations is conducted in a parallel mixed design fashion [44]. To be more specific, this approach can be considered an embedded design [45]. In this context, the qualitative data (SMS conversations) serves to elucidate the underlying processes that contribute to the effects observed in the quantitative data (the impact of the intervention). The outcomes of the intervention are subsequently combined with the qualitative data obtained from interviews with the fitness consultants, following a sequential mixed design approach [44]. This is executed as an explanatory design, where the qualitative data are used to provide explanations and additional insights into the results obtained from the intervention [45]. The mixed methods design also improves the construct validity of the study, that is, how well the findings from the analysis process reflect reality [46].

Owing to ethical considerations, the participating clients were kept anonymous to the researchers, and all client data were pseudonymized by the fitness consultants before they were handed over to the researchers. All participation was voluntary, and informed consent was obtained from all participants. SMS coaching was chosen because SMS is a widely adopted mobile function and an inexpensive mobile phone feature and has been proven to be effective in supporting behavior changes [32,38]. In addition, according to the Swedish Internet Foundation, Sweden is highly digitalized (92% own a smartphone [47], and 48% of the population uses a digital health service or mobile app on a weekly basis [48]). Thus, SMS is an inclusive method regardless of the type of mobile phone the participant owns, and the focus is not on the technical device but on the intervention itself [5].

### Participants

#### Clients

The analyses in this study were based on the data from 28 clients. We included 26 (n=9, 35% male and n=17, 65% female) clients in the quantitative analysis who completed all 3 data collections. Two clients were excluded from the quantitative analysis because we used only complete data: 1 had missing data for the SMS guide (but not the baseline data), and both had missing data for the waitlist. We used completion data to comprehend the impact of SMS coaching on participants who successfully completed the intervention. Thus, this analysis excluded clients who had dropped out or had incomplete data. As all 28 participants completed the intervention (including the 2 who had missing quantitative data), all SMS conversations were included in the qualitative analysis.

The mean age of the clients was 46.76 (SD 12.04; range 26-67) years (n=25), and their average weight was 106.50 (SD 19.41; range 73.30-158.80) kg (n=24), with an average waist circumference of 115.17 (SD 16.90; range 85-156) cm (n=24). Of the 26 clients, 20 (77%) wanted to lose weight by becoming more physically active and adopting healthier eating habits and 3 (12%) only mentioned wanting to change dietary habits. Most participants wanted to receive SMS weekly (13/26, 50%) or twice a week (10/26, 38%). Two clients requested more frequent SMS (ie, every second day).

The participating fitness consultants recruited clients for the study. Initially, 31 clients were invited to participate by their fitness consultants and started the study at T1. Of the 31 clients, 3 (3%) discontinued the study. The client sample corresponds to the overall client population.

#### Fitness Consultants

A total of 12 fitness consultants participated in this study. All 12 fitness consultants were women, their experience ranged from 1 to 28 years, and the majority had worked for >10 years. The fitness consultants’ ages were not always asked or stated in the interview but most were aged >35 years.

### Procedure

The study was cocreated with fitness consultants after a pilot study in 2019 that aimed to explore the usefulness of SMS coaching for FiV and its clients. The results of the pilot study were evaluated in a workshop where the fitness consultants provided their input based on their experiences. The pilot study resulted in an SMS guide with guidelines for SMS coaching and explicit SMS examples such as encouragements, reinforcements, and challenges, with MI and the COM-B model being the points of reference. This SMS guide was a starting point for the SMS intervention and was used to create a highly personalized coaching protocol. The SMS guide included personalization regarding frequency (eg, daily, every other day, or weekly), when to send the SMS (eg, the days or specific time points), and whether and when the clients were expected to respond. The SMS guide also regarded the target behavior (eg, “eat less sweets,” “exercise three days a week,” and “take a walk weekly”), an action plan for how to achieve the goal, and if the SMS could include pictures or links to web pages.

All fitness consultants were invited to participate in the study and were presented with a folder and a web page providing information about the study, informed consent contracts for clients, the SMS guide, and questionnaires for all assessment points. The information clearly stated that participation for all parties, both clients and fitness consultants, was voluntary; that they could discontinue their participation at any time; and that nonparticipation or discontinuation would not impact their continued contact or employment with FiV. No specific inclusion criteria for participation were stated by the researchers, and the fitness consultants were free to choose participants they deemed fit for SMS coaching. The web page also provided a randomization list where fitness consultants could randomize the recruited clients to start with either the SMS intervention or the waitlist (ie, the ABBA design).

As presented in Table 1, the intervention started (T1) with the SMS guide, self-rated questionnaires, and the basic health indicators (weight, waist circumference, and aerobic fitness). A similar protocol was followed at both time 2 (T2) and T3, where possible revisions could be made to the SMS guide. At T3, the fitness consultants also held a concluding talk with the clients about their experiences from the SMS coaching to allow the clients to give feedback and express their thoughts about their experiences. These concluding talks were not recorded or included in this study. The SMS conversations were with the consent from the clients pseudonymized and forwarded to the researchers.

After T3, the researchers interviewed the fitness consultants. The aim of these semistructured interviews was to learn about the fitness consultants’ experiences from the SMS coaching. The interviews were conducted in person or digitally via Zoom (Zoom Video Communications) or Facetime (Apple Inc) owing to the COVID-19 pandemic restrictions. The audio was digitally recorded with the consent of the interviewees and transcribed verbatim by professional transcribers.

### Materials

The materials included descriptions for the SMS guide and the quantitative data collected at baseline and after the SMS intervention and waitlist. Mean values were computed for all Likert scales.

#### Baseline

The baseline testing included the SMS guide and basic health indicators (weight [kg], waist circumference [cm], and aerobic fitness). Aerobic fitness was tested by measuring the liters of oxygen per minute (maximum oxygen consumption) that participants could consume while riding a stationary bike.

The lifestyle questionnaire was adapted from the WHO’s Global Physical Activity Questionnaire [42]. The questions assessed physical activity and sedentary habits (eg, “How much time do you spend in a regular week on physical activity [e.g., running, gymnastics, or ball sports] that raises your pulse?”). Answers for physical activity were given on a 7-point Likert scale ranging from 1 (0 minutes/no time) to 7 (>120 minutes/2 hours or >300 minutes/5 hours), depending on the intensity of the physical activity. Answers for dietary habits (eg, “How often do you eat vegetables or root vegetables?”) were given on a 5-point Likert scale ranging from 1 (never) to 5 (twice a day or more).

Lifestyle questions also include 3 self-rating scales for the participants’ perceived levels of overall health, physical activity, and dietary habits. The answers were given on a 10-point Likert scale ranging from 1 (very poor, sedentary, or needs improvement) to 10 (very well, regular physical activity, or does not need improvement).

The FiV questionnaire contained 5 areas. The first regarded basic demographics and background questions regarding sex, age, their first contact with FiV, and goal (containing a list of goals such as “well-being,” “lose weight,” or “become stronger” and a free text box to specify another goal).

The second part was an operationalization of the COM-B model [15]. A total of 16 items were construed to correspond to the components of the model: 4 items assessed capability (eg, “I know why it is important to [chosen behavior]”), 6 items assessed opportunity (eg, “I have enough time to [chosen behavior]”), and 6 items assessed motivation (eg, “I really want to [chosen behavior]”). All items were answered on a 7-point Likert scale ranging from 1 (do not agree at all) to 7 (totally agree).

The third part comprised 4 items regarding the client’s relationship with the fitness consultant (relationship with FiV, eg, “The Fitness consultant shows that they can see things from my perspective”). Items were answered on a 7-point Likert scale ranging from 1 (do not agree at all) to 7 (totally agree).

#### SMS Intervention

At the follow-up of the SMS intervention, the health testing, aerobic fitness, lifestyle questions, and FiV questionnaire from baseline were repeated. Furthermore, 3 items regarding the participants’ e*xperience of the SMS* intervention (eg, “I would like to continue with SMS coaching”) were added. The same 7-point Likert scale used for the FiV questionnaire, ranging from 1 (do not agree at all) to 7 (totally agree), was used.

#### Waitlist

For the waitlist, the health testing, aerobic fitness, lifestyle questions, and FiV questionnaire from baseline were repeated.

### Data Analysis

#### Quantitative Self-Report Data

Data from the SMS intervention regarding health testing, aerobic fitness, lifestyle questions, and SMS evaluation were used as outcome variables; changes indicated behavior change. All self-report data were analyzed to detect differences between the SMS intervention and the waitlist (ie, repeated measures or paired sample 2-tailed *t* tests), and differences indicated the effectiveness of the intervention. The α value was set to .05. Cohen *d* was used to estimate the effect size between the means. The rule of thumb is the cut-offs at 0.2, 0.5, and 0.8, which indicate a small, medium, and large effect size, respectively [49].

#### Qualitative Interview Data

The data were analyzed in accordance with the thematic analysis by Braun and Clarke [50] in 6 steps. An inductive analysis approach, wherein the semantic or explicit meaning of the interview was analyzed step by step to generate higher-order themes, was chosen. The initial coding was kept close to the data (ie, stayed close to the explicit meaning of the meaning-bearing unit). The analysis gradually distilled the themes into a comprehensive model of the data. This process led to the emergence of 3 aggregated themes that provide insights into the fitness consultants’ experiences and the lessons they learned from the SMS coaching. The first 2 steps were performed individually by 2 researchers to avoid bias, and the following 4 steps of refining, categorizing, and writing about data were a joint procedure.

#### SMS Conversations

SMS conversations were analyzed in 2 rounds. First, the SMS from the fitness consultants was analyzed in accordance with a deductive thematic analysis [50] to determine if and how their SMS followed the 3 components from the COM-B model, as the SMS examples provided in the SMS guide were developed using the COM-B [15]. Second, an inductive analysis was conducted on the clients’ SMS, similar to the procedure described as the inductive analysis approach for their behavior change process. Thus, the SMS conversations were analyzed both from an inductive and deductive approach, where the deductive analysis was used for answering the “what” of the COM-B model and the inductive analysis was used for answering the “how” this affected the clients’ behavior change process.

## Results

### Overview

Taken together, the SMS intervention showed positive effects on clients’ behavior changes, both with regard to the quantitative and qualitative data. The clients reported positive experiences from the SMS intervention and would have liked to continue with the SMS coaching (mean 5.86, SD 1.28; range 2.50-7.00; 25/26, 96%). The fitness consultants also wanted to continue the SMS coaching and considered it complementary to the ordinary work routine. The SMS conversations verified the results from both the questionnaires and interviews, showing how personalized SMS supported clients’ behavioral changes.

### Effects of the SMS Intervention on Health Data and Self-Rated Questionnaires

The paired sample *t* test showed that the participants’ waist circumference was reduced after the SMS intervention (t_20_=3.23, 95% CI 0.82-3.80; *P*=.004) and after the waitlist (t_22_=2.22, 95% CI 0.13-3.87; *P*=.04), in comparison with the baseline. Table 2 presents all descriptive data for the significant differences in the health data at baseline, SMS intervention, and waitlist.

The ratings for the lifestyle question, level of overall health, was improved after the SMS intervention in comparison with baseline (t_24_=−2.09, 95% CI −1.31 to −0.01; *P*=.047). An improvement was also observed in the clients’ level of dietary habits for the waitlist in comparison with baseline (t_23_=−2.66, 95% CI −1.96 to −0.24; *P*=.01). All differences had acceptable effect sizes (Cohen *d*), ranging from 0.42 to 0.70 (Table 2). No other differences were observed.

The paired sample *t* test of the FiV questionnaire showed that both capability (t_27_=−2.64, 95% CI −0.70 to −0.09; *P*=.01) and opportunity (t_27_=−2.29, 95% CI −1.61 to −0.03; *P*=.03) increased after the SMS intervention in comparison with the baseline. Ratings for the relationship with FiV were higher in the SMS intervention than in the waitlist (t_25_=2.59, 95% CI 0.06-0.52; *P*=.02). All differences had acceptable effect sizes (Cohen *d*), ranging from 0.43 to 0.51 (Table 3). No other significant differences were observed. Table 3 shows the descriptive data for the significant differences for capability, opportunity, and relationship with FiV.

**Table 2:** Mean, SD, and range for the significant differences (*P*<.05) in the physical fitness and lifestyle questions between the baseline, SMS, and waitlist condition.

		Values, mean (SD; range)	Values, n (%)
**Baseline**			
	Waist (cm)^a,b^	115.17 (16.90; 85.00-156.00)	24 (92)
	Level of overall health^c^	5.19 (1.91; 0.50-9.50)	26 (100)
	Level of dietary habits^d^	5.08 (2.23; 0.50-8.00)	25 (96)
**SMS intervention**			
	Waist (cm)^a^	112.57 (16.71; 84.00-155.00)	22 (85)
	Level of overall health^c^	5.88 (2.20; 2.00-10.00)	25 (96)
	Level of dietary habits	5.62 (2.06; 2.00-10.00)	25 (96)
**Waitlist**			
	Waist (cm)^b^	114.00 (17.85; 87.00-157.00)	24 (92)
	Level of overall health	5.86 (1.91; 2.00-9.50)	22 (85)
	Level of dietary habits^d^	6.15 (1.90; 1.00-9.00)	24 (92)

^a^Baseline versus SMS intervention: t_20_=3.23, 95% CI 0.82-3.80; *P*=.004; Cohen *d*=0.70.

^b^Baseline versus waitlist: t_22_=2.22, 95% CI 0.13-3.87; *P*=.04; Cohen *d*=0.69.

^c^Baseline versus SMS intervention: t_24_=−2.09, 95% CI −1.31 to −0.01; *P*=.047; Cohen *d*=−0.42.

^d^Baseline versus waitlist: t_23_=−2.66; *P*=.01, 95% CI −1.96 to −0.24; Cohen *d*=−0.54.

**Table 3:** Mean SD, range, and the internal consistency (Cronbach α) for the significant differences (*P*<.05) in the Fitness in Värmland (FiV) questionnaire (ie, capability, opportunity, and motivation and relationship with FiV) between the baseline, SMS intervention, and waitlist condition (n=26).

			Values, mean (SD; range)		Cronbach α
**Baseline**					
	Capability^a^	5.31 (0.88; 3.00-6.50)		.48	
	Opportunity^b^	5.36 (0.88; 3.50-7.00)		.48	
	Motivation	5.70 (0.74; 4.50-7.00)		.41	
	Relationship with FiV	6.55 (0.57; 5.00-7.00)		.49	
**SMS intervention**					
	Capability^a^	5.71 (0.74; 3.75-6.75)		.46	
	Opportunity^b^	5.68 (1.00; 3.00-7.00)		.66	
	Motivation	5.99 (0.76; 4.17-7.00)		.62	
	Relationship with FiV^c^	6.69 (0.56; 4.75-7.00)		.82	
**Waitlist**					
	Capability	5.59 (0.92; 3.75-6.75)		.70	
	Opportunity	5.54 (1.35; 2.17-7.00)		.88	
	Motivation	5.83 (0.86; 3.50-7.00)		.76	
	Relationship with FiV^c^	6.38 (0.74; 4.75-7.00)		.85	

^a^t_27_=−2.64, 95% CI −0.70 to −0.09; *P*=.01; Cohen *d=*0.50.

^b^t_27_=−2.29, 95% CI −1.61 to −0.03; *P*=.03; Cohen *d=*0.43.

^c^t_25_=2.59, 95% CI 0.06-0.52; *P*=.02; Cohen *d=*0.51.

### Results From the Interviews With Fitness Consultants After T3

The analysis of interviews with the fitness consultants revealed 3 major themes: supplement, lessons learned, and benefits.

#### Supplement

This theme regarded the fitness consultants’ overall view of the SMS coaching as a complementary method that facilitated the existing MI-based work method of individual counsels with the clients and consisted of 3 subthemes: continuity, easier feedback, and stressful to text*.*

Continuity assessed whether SMS coaching helped create a new way of maintaining continuous contact with the clients:

I have noticed that [SMS] are appreciated, and that it has deepened the contact and supported the clients continuously without them physically having to come to me. I have been able to be at hand in an easily accessible way.

P10

By providing detailed documentation about the client’s progress or challenges, the SMS conversations facilitated health talks by providing easier feedback:

It was easier now when I was doing the follow-up meeting to have these short notifications, so I knew what had happened between the [health talks]. That helped a lot.

P16

The third subtheme, stressful to text, was mainly regarded as a downside of introducing a new method to an already busy work schedule. Stressful to text also considered the actual texting; although some fitness consultants were used to texting, others developed their texting skills during the intervention, thus lessening the stress over time:

The SMS skills developed, and became increasingly easier.

P12

[I] think I started by writing down the SMS on paper. But not at the end [of the intervention].

P9

#### Lessons Learned

This theme regarded the lessons learned and experiences gained from the SMS coaching as a possible new work method and contained 2 subthemes: facilitators and challenges.

Facilitators regarded how the fitness consultants described the importance of having an explicit and agreed-upon plan for the SMS coaching with the client, that is, that both parties had the same expectations before the SMS coaching started with regard to frequency and content:

When you are in a [health talk] you can end up talking about anything from diets to relationships, it can become very unfocused. [With the SMS] it was focused, concrete.

P14

Facilitators can also consider setting reminders to send SMS. The subtheme, challenges, described the challenges of communicating through short texts, especially if the structure for the SMS coaching was unclear (“The challenge of formulating meaningful SMS” [P9]), or not knowing the recipient or their reactions. Another challenging aspect was that the fitness consultants felt that the SMS was repetitive (not varied enough).

#### Benefits

This third theme regarded how SMS coaching created value beyond the actual work method; for example, the flexibility of SMS coaching helped the fitness consultants better understand the clients’ everyday life as well as contributed to an improved relationship with the clients. Benefits included 3 subthemes: closer contact, flexibility for clients and consultants, and part of the client’s everyday life.

In general, the SMS coaching opened up for a closer contact with the clients, “It was eight weeks of SMS, so naturally you become close to [the client]. I mean, at midsummer when I was off work, I got ‘Have a nice midsummer!’” (P14), which was considered positive for motivation. Another aspect mentioned was that the SMS guide provided other types of questions helpful in getting to know the clients that they normally did not ask.

Fitness consultants also expressed how SMS coaching provided increased flexibility for both clients and the consultant, making it suitable for many different types of clients, some of whom may otherwise be hindered by the ordinary work process of regular individual health talks or group training. This can be illustrated by statements such as the following:

So his life situation. [SMS coaching] was perfect for him, both his work situation and his personality.

P9

In addition, the SMS coaching made the fitness consultants a part of their clients’ everyday lives and made clients more accountable and aware of their own role in the behavior change process:

[A client] told me that she felt that she was more honest now when sending SMS, and have really been honest about having a hard week.

P16

Another significant change was that the clients perceived it as if the fitness consultants visited the clients, in contrast to the previous arrangement where the clients visited the fitness consultants.

In summary, the analysis of the interviews generated 3 major themes. The first, supplement, can be summarized as the fitness consultants’ experiences from the intervention in general and how it affected their workload and regular work routine. The second, lessons learned, regarded the fitness consultants’ assessment from a professional perspective (ie, how the SMS coaching had affected the client in terms of motivation and actual behavior changes). The last theme, benefits, regarded how the intervention had promoted increased contact, flexibility, and continuity; created a new attitude among the clients; improved the relationship between the fitness consultants and the clients.

### Results From the SMS Conversations

The deductive analysis of the fitness consultants’ SMSs showed that all their SMSs can be categorized according to the COM-B model. Some SMSs are overlapping, that is, they can be categorized into 2 or even 3 of the COM-B concepts. Table 4 presents the main patterns.

The results from the inductive analysis of the clients’ SMSs revealed interesting patterns regarding their behavioral change processes. One pattern relates to failures and negative circumstances in the clients’ everyday lives, which were readily addressed when they emerged owing to the continuity of SMS coaching and seems to have contributed positively to the clients’ motivation and willingness to stick to the plan and not disrupt the behavioral change process. One of the clients stated the following:

With texting, I get to talk to you more often and have someone who keeps track.

Another finding was that the SMS coaching seems to have contributed positively to the participants’ motivation and mindset regarding healthy lifestyles, as illustrated in the following SMS examples:

I always think of the plate model.

Yes, I love the feeling I have right now with a lot of training and exercise; it has really changed me and my mindset.

The intervention also allowed for a more informal relationship between the fitness consultants and their clients and for the clients to get to know themselves better. Overall, the SMS conversations took on a friendly or even familiar tone, with some conversations ending with “hugs,” “good luck,” “wish you a happy birthday,” and so on. The SMS conversations also disclosed how the clients became aware of personal strengths and weaknesses, what worked well and what did not, and the type of support they needed:

Foresight and planning are probably the best.

But I notice that I eat far too irregularly, and far too many sandwiches.

In addition, the SMS conversations indicate that after some weeks, clients started to take responsibility for their own change work by taking initiatives and setting up goals on their own, as illustrated in the following quote:

It feels good. I have brought [training clothes], so I can take an hour-long walk right after [work] tonight. The plan is to take a walk on Sunday as well! Tonight, it will be a few kilometers when I get to [city]. A total of 17.5 kilometers last week. [I will] try to [walk] 20 kilometers this week.

This also demonstrates the client’s belief in their own ability to change their lifestyle and achieve the desired behavior. Overall, the SMS conversations show an improvement over time in the clients’ capabilities and opportunities (ie, what to do and when) and their motivation (ie, how they feel and think about their behaviors).

**Table 4:** Overview of the example quotes from the deductive analysis of SMSs according to the Capability, Opportunity, Motivation–Behavior (COM-B) model (n=28).

Themes and descriptions			Example quote
**Capability**			
	SMS addressed the client’s belief in their own capacity to perform the desired or targeted behavior, often in terms of strengthening the client’s belief in succeeding.	*You know what you need to do and you know what you can do.* *What improvements have you experienced from the exercises that you have performed?*	
**Motivation**			
	SMS that encouraged or positively reinforced a behavior.	*Great, you have already been out [on a walk] today, and have also made plans for tomorrow.* *You work really hard and plan ahead to achieve your goal. Great, keep up the good work.*	
	SMS that focused on feelings and attitudes.	*It feels good to you. You think before you make a choice.* *Do you get positive feelings from the training?*	
	There were also SMSs that included tips (eg, exercises, podcasts, and recipes).	*Our physiotherapist has made a new sheet with exercises mainly for the stomach and back. Sending a picture of it here.* *Here you get a new suggestion for smoothies (image of a recipe for smoothies).*	
**Opportunity**			
	SMS that helped spotting opportunities to perform the desired or targeted behavior. These SMSs included questions about the plan for the day, week, or weekend.	*How does your plan look for the weekend when it comes to food and physical activity?*	
	SMS can be reminders for the agreed plan and tips and advice.	*Remember the plate model!* *You can divide your walks into several 10-minute sessions spread over a day.*	

## Discussion

### Principal Findings

This study found that personalized SMS coaching is effective in supporting behavior changes grounded in the COM-B model and that both fitness consultants and clients had positive experiences and wanted to continue using SMS coaching. The SMS coaching resulted in reduced waistlines and improved overall health. The results also show that personalized SMS coaching enhances personal relationships (human connection) and strengthens the behavior change process (added value).

**Figure 1:**
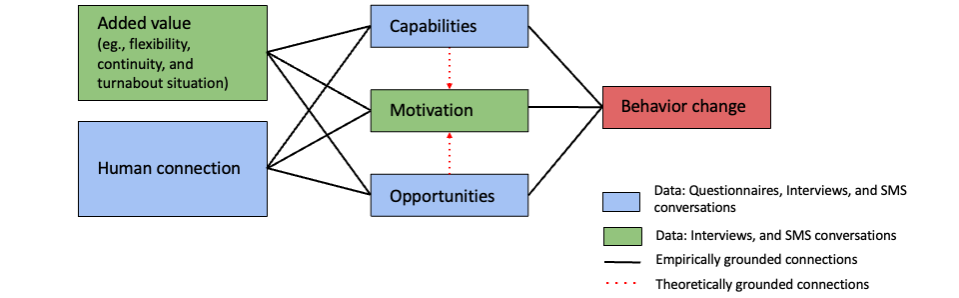
The results from all 3 data sets (ie, self-reported questionnaires, interviews, and SMS conversations) and how the personalized SMS contributed to behavior changes.

The results from all 3 data sets (ie, self-reported questionnaires, interviews, and SMS conversations) and Figure 1 show how personalized SMS contributes to the clients’ behavior changes. The connections depicted in Figure 1 pertain to the findings of this study, but they do not entirely align with the interactions and directions outlined in the COM-B model by Michie et al [15].

Personalized SMS coaching seemed especially effective in empowering the clients, as shown in the quantitative analysis where the clients’ capabilities and opportunities increased during the intervention. Thus, the intervention using the COM-B model as a point of reference [15] helped the clients feel more capable of performing the behavior and finding opportunities to do so. Fitness consultants noticed that the clients became more accountable for their behavior change process (eg, taking more of their own initiative and changes in attitudes toward behavior changes). The analysis of the SMS conversations validated this result, with several examples of how the clients displayed more initiative and willingness even under suboptimal circumstances.

Furthermore, the findings show that personalized SMS coaching complements the existing work methods at FiV and enriches the relationship between fitness consultants and their clients. The intervention established continuity and flexibility, facilitating a closer rapport between the health talks. As Figure 1 illustrates, the value of personalized SMS coaching is referred to as added value and includes aspects such as the complementary method as well as continuity, flexibility, and the turnaround situation created by the SMS coaching.

### Strengths and Limitations

An obvious strength of this study is the use of a naturalistic sample, which facilitates the exploration of user experience and environment [21,30]. Another strength is the use of 3 types of data (longitudinal self-reported quantitative data from questionnaires and health assessments, qualitative interviews with fitness consultants after the interventions, and pseudonymized qualitative SMS conversations), which enabled us to understand the effects of the SMS coaching as well as follow the process and explore the experiences of the participants. This triangulation allowed for indirect validation of the results and a deeper understanding of the process.

A possible limitation is the use of an unvalidated COM-B instrument. As Table 3 shows, the internal consistency, as assessed by Cronbach α, was low at baseline for capability, opportunity, and motivation but increased over time. However, internal consistency can be affected by small sample size or seen as an indication of the clients’ understanding of the items, or in this case, their resources. Hence, the initial low and subsequently increasing levels of internal consistency might reflect the learning curve of the participants rather than a psychometric issue. The data triangulation supports this explanation, as both the interviews and SMS conversations point to the development of clients and their behavior changes. Therefore, the internal consistency of items (Cronbach α) is not entirely an accurate measure in this context. Low internal consistency can be a reliability problem that needs to be addressed in future studies.

### Comparison With Similar Research

The results of this study are in line with previous studies that show how strengthening a person’s capability is an important driver for behavior change [19,20,29,36]. This is also in line with several studies that have testified that personalized SMS in terms of demographics, motivation, and requested SMS frequency support behavior changes [29,32,38].

An aspect of the added value that emerged during the interviews was the importance of learning about the clients’ everyday life and the turnaround created by the SMS coaching. The clients no longer came to a meeting with the fitness consultants, but the fitness consultants became a “virtual visitor” of the clients, which created a different dynamic in their relationship. A turnaround that mainly seems to be related to the continuity and closeness of the SMS intervention created. This turnaround situation aligns with the findings of Bäccman et al [51], who explored the user experience of an automatic shower, where the users, both personnel and clients, experienced a shift in power and control when using the automatic shower. The clients’ perception of the whole shower situation changed even if most aspects apart from the actual shower remained the same (eg, getting to the shower, dressing and undressing, and getting help to dry). This small change in perception was essential for the clients’ sense of control and perceived independence. These results demonstrate the importance of user involvement in the context of use where unexpected aspects of using digital technology can be discovered [52].

### Explanations of Findings

As illustrated in Figure 1, human connection—and its importance for the participants in this study—can be observed in all 3 data sets. The questionnaires showed that the SMS intervention positively contributed to the clients’ relationship with the fitness consultants in comparison with the baseline and waitlist. This improved relationship was also articulated by the fitness consultants and emerged as a theme from the analysis of the interviews. In the SMS conversations, human connection was manifested as an increasingly familiar and easy tone between the fitness consultants and their clients. This result can be compared with the importance of the therapeutic alliance in psychotherapy, where the bond between the therapist and the client is a well-known predictor of the therapeutic outcome [40,53]. Thus, forming a bond with the client seems to be an important factor, regardless of the setting or type of professional relationship. However, it is still unclear whether the relationship is a mediating or moderating aspect of the SMS coaching [54].

The high level of personalization corresponds with Spark et al [37], who found that DHI should include “an element of human connection to foster ongoing participant satisfaction and accountability.” A similar result was reported by Godino et al [36], who found that SMS was most effective when combined with monthly phone calls. It is important to consider whether the human connection is sufficient to support behavior change on its own. For example, a future study can use the following two conditions: (1) personalized SMS with BCTs similar to this study and (2) only personalized coaching where the SMS is mere encouragement and reminders, thus not actually providing BCTs. If the effect of these 2 conditions is the same, personal contact can be concluded to be effective, regardless of the content or the competence of the coach. If so, personalized SMS could be used more freely and would not require personnel trained in either MI or BCT, making it easier to scale up.

In contrast to several other studies [19,20,29,36,38], we did not observe any differences in motivation between the conditions. However, as Figure 1 illustrates, the qualitative data, both the interviews and SMS conversations, imply that the SMS intervention increased motivation. An explanation for this discrepancy may be that client motivation was already high at baseline, making incremental changes difficult to detect. Thus, the difference observed is a reflection of what is known as “selection effects,” where the motivation level among the participants was higher than that of the general population, which can make it more challenging to detect changes. This is akin to the concept of “restriction of range” [55]. Another related explanation may be that the questionnaire is unable to detect changes, thus not being sensitive enough.

A third plausible explanation for the inconsistency between the quantitative and qualitative data may be that motivation was something that occurred in the interaction between the fitness consultant and the clients, making it more difficult to assess quantitatively at a specific time (ie, at T2 or T3). In other words, the noted motivation was a product of the intervention mode, that is, the fitness consultants’ experience with MI and personalized SMS [16,20,37]. Hence, it is essential to recognize that the emotional and cognitive facets of motivation are dynamic states shaped by the actions and conditions fostered by the design of a DHI, rather than being tied to a specific BCT. As noted, personalized SMS is “a mode of delivery rather than a BCT” [16]. Similarly, learning is influenced by the feedback process, where the behavior is reinforced by the observed reactions from the surrounding [11,12], again pointing toward the ever-changing possibility of motivation. However, this separation of the “the what” (eg, BCT) and “the how” (eg, personalized SMS or MI) is merely a theoretical separation, and the practical aspects are much more entwined [40].

### Implications and Future Studies

This study supports the studies that claim that DHI needs to support social interactions [20,37]. Here, we used personalized SMS grounded in the COM-B model and personalized SMS to the client’s needs as well as used MI as a mode for delivery. To fully understand the effective aspects of SMS and any DHI, it is important to further investigate how human connections, personalization, and BCTs interact during a DHI to optimize its potential for behavior change. So far, it seems as if real or perceived human connection is an essential part of DHI.

User involvement is necessary to fully understand the effects of DHI. The results from the studies pressing for user involvement and the human connection of personalization [21,29,30] were obtained after the completion of our study, which validates the results of our study. Not involving end users in the process will likely result in more digital solutions that no one uses.

### Conclusions

This study makes a significant contribution to the literature because it uses (1) personalized SMS, which is theoretically grounded (ie, COM-B) and tailored to the client’s specific needs; and (2) 3 types of data, allowing for indirect validation of the results and a deeper understanding of the process. We have not come across any other study with this design.

The main findings of this study show that personalized SMS coaching is effective in supporting behavior changes. This is promising because SMS is an affordable digital solution that many already have access to [5,32,33,38,47]. The fact that FiV has implemented personalized SMS coaching as an ordinary work method after this study bears witness to the scalability and functionality of personalized SMS. In other words, personalized SMS coaching is scalable and accessible to a large portion of the population, and intricate digital solutions to support behavior change are not always necessary.

## Abbreviations

BCT: behavior change technique
COM-B: Capability, Opportunity, Motivation–Behavior
DHI: digital health intervention
FiV: Fitness in Värmland
MI: motivational interviewing
SMART: specific, measurable, attainable, realistic, timely
T1: time 1
T2: time 2
T3: time 3
WHO: World Health Organization
